# Identification of reproducible drug-resistance-related dysregulated genes in small-scale cancer cell line experiments

**DOI:** 10.1038/srep11895

**Published:** 2015-07-15

**Authors:** Lu Ao, Haidan Yan, Tingting Zheng, Hongwei Wang, Mengsha Tong, Qingzhou Guan, Xiangyu Li, Hao Cai, Mengyao Li, Zheng Guo

**Affiliations:** 1 Department of Bioinformatics, Key Laboratory of Ministry of Education for Gastrointestinal Cancer, School of Basic Medical Sciences, Fujian Medical University, Fuzhou, 350108; 2 Biomarker Technologies Corporation, Beijing, 101300; 3 College of Bioinformatics Science and Technology, Harbin Medical University, Harbin, 150001.

## Abstract

Researchers usually measure only a few technical replicates of two types of cell line, resistant or sensitive to a drug, and use a fold-change (FC) cut-off value to detect differentially expressed (DE) genes. However, the FC cut-off lacks statistical control and is biased towards the identification of genes with low expression levels in both cell lines. Here, viewing every pair of resistant-sensitive technical replicates as an experiment, we proposed an algorithm to identify DE genes by evaluating the reproducibility of the expression difference or FC between every two independent experiments without overlapping samples. Using four small datasets of cancer cell line resistant or sensitive to a drug, we demonstrated that this algorithm could efficiently capture reproducible DE genes significantly enriched in biological pathways relevant to the corresponding drugs, whereas many of them could not be found by the FC and other commonly used methods. Therefore, the proposed algorithm is an effective complement to current approaches for analysing small cancer cell line data.

In experiments for comparing gene expression profiles between two types of cancer cell lines which are respectively resistant and sensitive to a particular drug, researchers usually generate only two or three technical replicates for each type given that there is no biological difference between technical replicates for a particular clonally derived cell line. Typically, in these small datasets, genes are selected as differentially expressed (DE) genes if their fold changes (FCs), computed as the ratios of the average gene expressions of the genes between the two types of cell lines, are larger than a arbitrarily determined cut-off value. Obviously, genes that are highly expressed in both cell lines can hardly reach large FCs, and thus the FC approach tend to miss them even if their absolute expression level differences between the two types of cell lines are rather large^1^. Besides, genes with low expression levels in both cell lines may reach large FCs simply due to large measurement variations, whereas the FC approach lacks of statistical control to reduce such false discoveries. Significance Analysis of Microarrays^1^ (SAM) and the Rank Product^2^ (RP) methods have also been applied to analyse small datasets from cell line experiments^3^,^4^. However, similar to FC, they are both biased towards the identification of genes with large FCs between two types^5^,^6^,^7^,^8^,^9^.

Compared with genes expressed at low levels, genes with high expression levels are more likely to participate in some more conserved pathways with important biological significance, such as RNA processing, metabolism, and membrane trafficking^10^. To increase the power of detecting genes with high expression levels, the Average Difference (AD) method have been proposed, which ranks genes by the difference in average expression levels between two groups of samples^3^. In contrast to FC, SAM and RP, AD tends to identify genes that are highly expressed in both cell lines with large absolute differences in expression levels and may miss genes expressed at low levels in both cell lines, even if their FCs are truly large. In response, the Weighted Average Difference (WAD) method^3^ is proposed to rank genes using the relative average signal intensity as the weight to compute the gene expression ratio between two types. However, the WAD method is limited by uncertainties in weighting. Furthermore, both AD and WAD lack statistical control.

In the present study, for small datasets that include several technical replicates for each cell line, we considered every pair of resistant-sensitive technical replicates as an experiment and every two experiments without overlapping samples as independent experiments. Then, we proposed an algorithm to rank genes according to their absolute expression differences or FCs in each pair of resistant-sensitive cell line experiment and identified significantly reproducible DE genes between every two independent experiments through reproducibility evaluation^11^. The algorithm was comprehensively evaluated using four microarray datasets for drug-resistant and drug-sensitive cancer cell lines.

## Results

### The reproducibility-based PD ranking method

**Figure 1:**
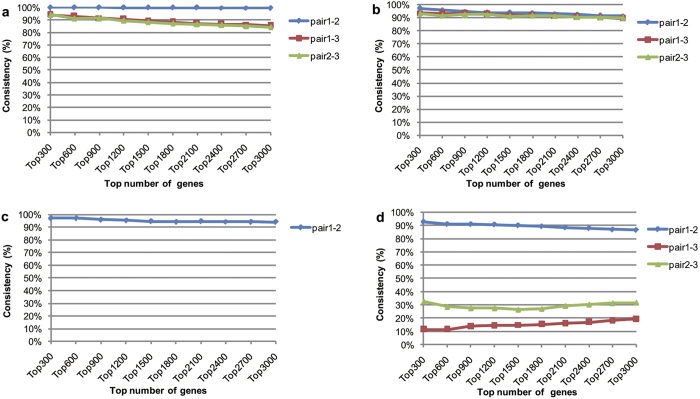
The evaluation of consistency scores between every two independent pairs. The consistency scores of top *n* (*n *= 300, 600,……3000) genes ranked by PD between every two independent pairs were evaluated in the CP70/A2780 dataset (**a**), the MDA-MB-231dataset (**b**), the LCC2/MCF7 dataset (**c**) and the HCT116 dataset (**d**). Consistency(%) ranged from 0%-100%, top 300 (top 600, etc.) were the top number of genes of two independent pairs ranked by PD. pair1-2, pair1-3, pair2-3 were the comparisons of reproducibility between pair 1 and pair 2, pair 1 and pair 3, pair 2 and pair 3 in each dataset, respectively.

In each of the four datasets, referred to as CP70/A2780, MDA-MB-231, LCC2/MCF7 and HCT116, we ranked genes according to the Pairwise Difference (PD) of expression levels in every pair of resistant and sensitive replicates, and then evaluated the dysregulation direction consistency score of the top *n* (*n *= 300, 600,……3000) genes between every two independent pairs without overlapping samples (see *Methods* for details). In the CP70/A2780, MDA-MB-231, LCC2/MCF7 datasets, the consistency scores of the top *n* (*n *= 300, 600,……3000) genes sorted from every two independent pairs were all significantly higher than expected by chance (binomial test, all *p *< 2.2E-16)(Fig. 1a–c). In the HCT116 dataset, the top *n* (*n *= 300, 600,……3000) genes between pair 1 (MEXP:179508 VS MEXP:179487) and pair 2 (MEXP:179509 VS MEXP:179486) were significantly consistent (binomial test, all *p *< 2.2E-16), suggesting that there were reproducible differential expression signals between the two independent pairs (Fig. 1d). However, the top *n* (*n *= 300, 600,……3000) genes in another independent pair, pair 3 (MEXP:179507 and MEXP:179488), were inconsistent with the top *n* (*n *= 300, 600,……3000) genes ranked by PD in either pair 1 or pair 2, with all the consistency scores below 33%. The results suggested that unreliable measurements may be present in one or two samples of pair 3. Therefore, we excluded pair 3 and used only pair 1 and pair 2 for the subsequent analyses in this study. Detailed information about the independent pairs selected in each dataset is provided in Table 1.

The consistency score between two gene lists with reproducible differential expression signals gradually decreased with increasing *n*, indicating the importance of optimising the length of the initial step. For example, in pair 1 and pair 2 of the HCT116 dataset, when *n* was set to 300 (i.e., the top 1 to 300 genes were included), the overlapping number was 147. Out of these 147 genes, 136 genes had the same dysregulation directions in the drug-resistant cell compared with the drug-sensitive cell in the two independent experiments; the consistency score was 92.52%. When *n *= 1200, although the consistency score for the overlapping 750 genes reached 90.53%, which was above the acceptability threshold of 90%, the consistency score between the genes in the 4th block (that is, the top 901 to 1,200 genes) was only 89.59%. Thus, at a certain consistency threshold, the consistency score between genes at the top of one block of the two gene lists was higher than the consistency score between genes at the bottom of the same block at the same step. Thus, when applying the reproducibility-based PD ranking algorithm to identify DE genes, a large initial step would allow genes with a low consistency score at the bottom of the block exceed the consistency threshold to become candidate DE genes and increase the probability of false positives. By contrast, if the initial step is too short, the number of overlapping genes between two gene lists could be too small to reach stable consistency scores, and the search process will likely terminate prematurely. As a result, an initial step that included 300 genes was adopted in the subsequent analyses. In the last but one section , we will further analyse the influence of parameter selection on the performance of the algorithm in detail.

Using the reproducibility-based PD with an initial step of 300 and a consistency threshold of 90%, 6,366, 2,031, 2,928 and 1,057 DE genes were detected for the CP70/A2780, MDA-MB-231, LCC2/MCF7and HCT116 datasets, respectively. These DE genes were enriched in 9, 11, 19 and 12 KEGG pathways with an FDR less than 5%, respectively (Supplementary Table S1–S4). Because a DE genes list can be significantly enriched in pathways only when it contains sufficient real DE genes^12^,^13^, these results suggest that the reproducibility-based PD algorithm can detect reliably DE genes in small datasets including only two or three technical replicates.

To prove this, we did random experiments based on the dataset CP70/A2780 as an example. From the background genes (i.e., all the measured genes) in this dataset, we randomly selected a set of genes with the same number of DE genes detected by the reproducibility-based PD method with an initial step of 300 and a consistency threshold of 90%, and then did the enrichment analysis to count the number of pathways that were significantly enriched with the randomly selected genes at the same FDR level of 5%. This random experiment was repeated 1,000 times. The result showed that 97% of the 1,000 random experiments could not identify any significant pathway and the average of the number of significant pathways of the 1,000 random experiments was 0.043, whereas the number of the pathways significantly enriched with DE genes detected by the reproducibility-based PD method was 9. None of the 1,000 random experiments detected more significant pathways than the reproducibility-based PD method, indicating that the random probability of observing no less than the number of significant pathways detected by the reproducibility-based PD method was less than 0.001. In addition, we randomly replaced a certain proportion of the DE genes detected by the reproducibility-based PD method with the same number of genes randomly selected from the background genes (excluding genes in the DE genes list), and then did the enrichment analysis. With a mixture of 75% genes from the DE genes list and 25% genes from the background, averagely only 4.32 significant pathways could be detected in 1,000 random experiments and the number decreased to 0.62 when the proportion of random genes increased to 50%. The results showed that the number of pathways enriched by genes decreased greatly as the proportion of random genes (i.e., false DE genes) in the DE genes lists increased. It suggested that most of the DE genes detected by the reproducibility-based PD method were reliable.

We also need to notice that the drug-resistant cancer cell lines in the four datasets were induced to resistance by repeated exposure to increasing concentrations of particular drugs, and thus some genes differentially expressed between drug-sensitive cancer cell lines and drug-induced resistant cancer cell lines may just represent drug-induced transcriptional changes that may be irrelevant to drug resistance mechanisms^14^,^15^. Nevertheless, for each dataset, many of the biological pathways significantly enriched with the DE genes detected by the reproducibility-based PD method are related to drug sensitivity. For example, the DE genes detected by the reproducibility-based PD method for the CP70/A2780 dataset of cisplatin resistant-sensitive cell lines were significantly enriched in biological pathways which are known to be related to drug cisplatin sensitivity, such as cell cycle, P53 signalling pathway, ubiquitin mediated proteolysis and protein processing in endoplasmic reticulum (see Supplementary Table S1). The target of cisplatin is thought to be DNA and the formation of cisplatin-DNA adducts block DNA replication and transcription, leading to cell cycle arrest and apoptosis^14^. P53 is involved in DNA damage signaling and many studies^14^,^16^,^17^,^18^ have reported that the activity of P53 is related with the sensitivity of cancer cell to cisplatin. The relationship between ubiquitin mediated proteolysis and DNA damage response is close^19^ and cisplatin can activate the pathway. It has been suggested that ubiquitin- proteasome genes as targets for modulating cisplatin sensitivity^20^. Cisplatin is also shown to cause apoptotic caspases through the endoplasmic reticulum stress pathway^21^,^22^. In supplementary Table S2–S4, we have listed some references which provided evidence of the corresponding drug sensitivity relevance of the biological pathways enriched by DE genes detected by the reproducibility-based PD method for the other three datasets.

In addition, we did enrichment analysis of DE genes obtained by the reproducibility-based PD by using the GO-function tool^23^ based on Gene Ontology^24^ with an FDR control (<5%). Similarly, many meaningful biological processes were identified (see supplementary Table S5–S8).

### Comparison of the reproducibility-based PD with FC, SAM and RP

**Figure 2:**
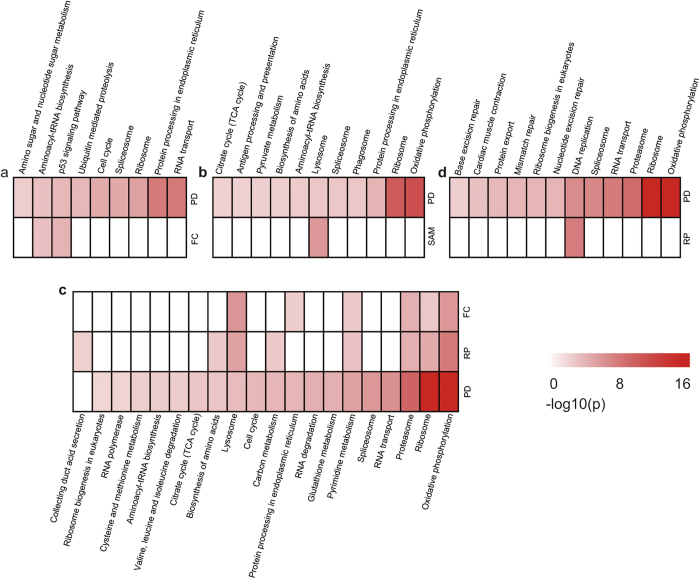
The comparisons of enrichment analysis. DE genes identified by reproducibility-based PD, FC, SAM or RP in the CP70/A2780 dataset (**a**), the MDA-MB-231 dataset (**b**), the LCC2/MCF7 dataset (**c**) and the HCT116 dataset (**d**) were used in the KEGG enrichment analysis. The reproducibility-based PD detected DE genes with an initial step of 300 and a consistency threshold of 90% and FC selected the same number of top genes as the reproducibility-based PD, not by a cut-off value. SAM detected DE genes with a 10% FDR and RP was with a statistical control level of *p *< 0.05 and 1000 permutation. All *p* values of the KEGG pathway were adjusted by Benjamini and Hochberg in four datasets (*p *< 0.05). -log10(*p*) was used to generate the heat map.

In the CP70/A2780, MDA-MB-231, LCC2/MCF7 and HCT116 datasets, we detected 3,433, 1,523, 1,680 and 126 DE genes, respectively, by conventional FC when the FC cut-off value was 1.5. Only the DE genes of the LCC2/MCF7 dataset were enriched in one KEGG pathway with the same FDR control of 5% as the reproducibility-based PD. When the cut-off value was changed to 2, only the DE genes selected from the MDA-MB-231dataset were enriched in two KEGG pathways. Moreover, when using FC to select the same number of top-ranked genes as the reproducibility-based PD, the genes selected in the four datasets were enriched in two, zero, six and zero KEGG pathways (5% FDR) (Fig. 2a,c), respectively. The rare functional enrichment results of the DE genes detected by FC with a simple cut-off suggest that this method may yield a high false-positive rate and/or miss many functionally important DE genes^12^,^13^ .

**Figure 3:**
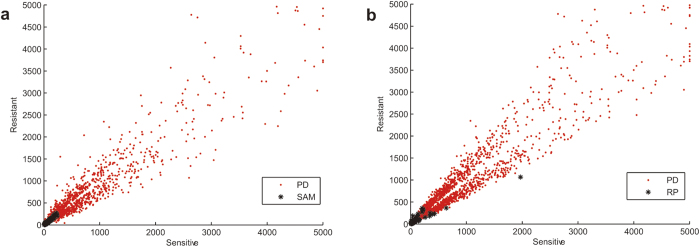
The distributions of DE genes exclusively detected by PD, SAM or RP in the CP70/A2780 dataset. (**a**–**b**) The average expression levels of DE genes in resistant replicates and sensitive replicates were plotted. PD, SAM and RP represent the method to identify DE genes. The average expression level above 5,000 was set to 5,000.

A total of 5,780, 2,785, 4 and 0 DE genes were detected by SAM with a 10% FDR in the CP70/A2780, MDA-MB-231, LCC2/MCF7and HCT116 datasets, respectively. These results demonstrate that SAM has low power for small datasets with only two or three technical duplicates. Only DE genes identified in the MDA-MB-231 dataset were enriched in one KEGG pathway (5% FDR) (Fig. 2b). Therefore, SAM missed many drug resistance-related pathways that were detected by the reproducibility-based PD. Although the numbers of DE genes detected by SAM and PD were comparable for the CP70/A2780 and MDA-MB-231datasets, approximately 28–45% of the DE genes detected by the reproducibility-based PD were not identified by SAM. In addition, approximately 25–30% DE genes detected by SAM were not found by the reproducibility-based PD. Compared with the DE genes exclusively detected by the reproducibility-based PD, the DE genes exclusively detected by SAM had very low expression levels in both the drug-resistant and sensitive cells. For example, in the CP70/A2780 dataset, the average expression levels of DE genes only detected by SAM were 31.06 and 30.73 in the drug-resistant and drug-sensitive cells, respectively. By contrast, the average expression levels of the DE genes detected only by the reproducibility-based PD were 813.11 and 833.82 (Fig. 3a). Similar results were observed in the MDA-MB-231 dataset (see Supplementary Figure S1a). These results demonstrate that, similar to FC, SAM is biased toward the identification of genes expressed at low levels as DE genes.

With 10% FDR control, 1,440, 690, 473 and 72 DE genes were detected by RP with 1,000 permutations in the CP70/A2780, MDA-MB-231, LCC2/MCF7 and HCT116 datasets, respectively. However, only DE genes selected in the LCC2/MCF7 dataset were enriched in one KEGG pathway with 5% FDR control. To obtain a number of DE genes selected by RP comparable to that obtained by the reproducibility-based PD, we used a statistical control level of p < 0.05, which resulted in the detection of 3,136, 1,956, 2,928 and 2,409 DE genes in the four datasets, respectively. Only genes identified in the LCC2/MCF7 and HCT116 datasets were differentially expressed in eight and one KEGG pathways, respectively (5% FDR) (Fig. 2c,d). Therefore, RP also failed to identify many drug resistance-related pathways that could be detected by the reproducibility-based PD. In the four datasets, we observed that approximately 9–75% of DE genes detected by the reproducibility-based PD were not identified by RP and 21–55% of the DE genes detected by RP were not identified by the reproducibility-based PD. Compared with the DE genes exclusively detected by the reproducibility-based PD, the DE genes exclusively detected by RP had low expression levels in both drug-resistant and drug-sensitive cells. For example, in the CP70/A2780 dataset, the average expression levels of DE genes only detected by RP were 46.05 and 49.28 in the drug-resistant and drug-sensitive cells, respectively. By contrast, the average expression levels of DE genes only detected by the reproducibility-based PD were 762.94 and 761.30 (Fig. 3b). Similar results were obtained for the other datasets (see Supplementary Figure S1b–d). These results demonstrate that, similar to FC and SAM, RP is also biased towards the selection of genes expressed at low levels as DE genes.

### The influence of parameter settings in the reproducibility-based PD

First, we evaluated the effect of the consistency threshold (CT) setting on the performance of the reproducibility-based PD with a fixed initial step of 300. When the CT was set to 85%, then 6,788, 2,842, 4,893 and 1,511 DE genes were identified in the CP70/A2780, MDA-MB-231, LCC2/MCF7 and HCT116 datasets, respectively, representing increases of 6.63%, 39.93%, 67.11% and 42.95% compared to a CT value of 90%. When CT was increased to 95%, the number of DE genes selected in the four datasets was 5,144, 758, 1,139 and 326, respectively, representing decreases of 19.20%, 62.68%, 61.10% and 69.16% compared to a CT of 90%. Notably, for all the four datasets, although the number of DE genes varied with the CT, the identity of the biological pathways enriched by DE genes was rather stable. Of the 9, 11, 19 and 12 pathways significantly enriched for DE genes detected using a CT of 90% (FDR < 5%), all were also significantly (FDR < 5%) or tentatively (p < 0.05) enriched for DE genes detected with a CT of 85%. Moreover, 9, 7,11 and 9 pathways were significantly (FDR < 5%) or tentatively (p < 0.05) enriched for DE genes detected with a CT of 95%. Additionally, in the LCC2/MCF7 and HCT116 datasets, a CT of 95% yielded so few DE genes that few enriched biological pathways could be detected with a FDR < 5%. In such a situation, the CT should be reduced (e.g., to 85%) to more accurately capture the global alterations in gene expression between the two conditions^25^,^26^. Although the false-positive rate tended to increase as the CT decreases, we still detected biologically meaningful pathways. Indeed, the pathway enrichment analysis was robust against a certain rate of false positives^13^. For example, in the data for the 5-FU-resistant and 5-FU-sensitive colorectal cancer cell-lines HCT116, 1511 DE genes were detected when the CT was 85%. These genes were enriched 17 KEGG pathways at a 5% FDR. Many of these pathways, such as DNA replication^27^, base excision repair^28^ and mismatch repair^28^,^29^, are relevant to 5-FU resistance.

Next, we fixed the CT at 90% and evaluated the effect of the size of the initial step. Because the initial step should not be too large, as explained in the first section, we assessed two smaller initial steps of 200 and 100 and compared the results to those obtained using an initial step of 300. With an initial step of 200, the number of DE genes selected in the CP70/A2780, MDA-MB-231, LCC2/MCF7 and HCT116 datasets was 6,302, 2,005, 2,589 and 841, respectively, representing decreases of 1.01%, 1.28%, 11.58% and 20.44% compared with the results obtained using an initial step of 300. With an initial step of 100, the number of DE genes selected in the four datasets was 6,036, 1,494, 1,615 and 941, respectively, representing decreases of 5.18%, 26.44%, 44.84% and 10.97% compared with the results obtained with an initial step of 300. Thus, a small initial step may terminate the search process too early, yielding an unstable consistency score between two short gene lists. Nevertheless, we observed that the biological pathways enriched for DE genes selected with the three different initial steps highly overlapped. Among the 9, 11, 19 and 12 pathways significantly enriched (FDR < 5%) for DE genes detected with an initial step of 300 for the four datasets, respectively, 9, 11, 19 and 9 pathways were also significantly (FDR < 5%) or tentatively (p < 0.05) enriched for DE genes detected with an initial step of 200, and 9, 11,17 and 12 pathways were significantly (FDR<5%) or tentatively (p < 0.05) enriched for DE genes detected with an initial step of 100.

Taken together, these results demonstrated that the CT and initial step value had a large influence on the number of genes selected as DE genes but less influence on the functional analysis results. In other words, the enrichment pathways were rather stable for the results of the reproducibility-based PD with different parameter settings.

### The reproducibility-based PFC ranking method

Because the conventional FC lacks statistical control, we transformed FC values into Pairwise Fold Change (PFC) values based on the reproducibility evaluation. Similar to the results for the reproducibility-based PD, in three of the four datasets (not HCT116), the genes sorted by the reproducibility-based PFC from every two independent pairs were highly consistent when *n* increased from 300 to 3000 (binomial test, all *p *< 2.2E-16) (see Supplementary Figure S2). In the HCT116 dataset, the top *n* (*n *= 300, 600,……3000) genes ranked by PFC in pair 3 (MEXP:179507 and MEXP:179488) were inconsistent with the top *n* (*n *= 300, 600,……3000) genes ranked by PFC in either pair 1 or pair 2. Detailed information about the independent pairs selected by the reproducibility-based PFC in each dataset is provided in Supplementary Table S9.

Evaluation of the consistency of the top 300 genes sorted by PD between each two independent pairs selected by PFC revealed that they were all significantly consistent (binomial test, all *p *< 2.2E-16) and vice versa. In the four datasets, we detected 6,259, 2,933, 3,627 and 998 DE genes by the reproducibility-based PFC with a CT of 90% and an initial step of 300. The DE gene lists for the CP70/A2780, MDA-MB-231, LCC2/MCF7and HCT116 datasets were enriched in two, four, seven and one KEGG pathways, respectively, with FDR < 5% (see supplementary Table S10). As shown in Supplementary Table S1–S4 and S10, although most of the pathways enriched for DE genes detected by the reproducibility-based PFC were also detected by the reproducibility-based PD for each dataset, some enrichment pathways were unique to the reproducibility-based PFC. For example, in the MDA-MB-231 dataset, DE genes identified by the reproducibility-based PFC were enriched in the terpenoid backbone biosynthesis pathway, a core synthetic route for paclitaxel^30^,^31^ that was missed by the reproducibility-based PD. Thus, the reproducibility-based PFC could provide additional, valuable information in small-scale cancer cell line datasets.

In conclusion, the reproducibility-based PD and PFC can identify significant, reproducible DE genes and functions in small-scale cancer cell line datasets. The reproducibility-based PD and PFC can be combined to detect genes with different expression levels and establish a more accurate report of differences between two types.

## Discussion

For identifying DE genes in small-scale cell line datasets, the conventional FC lacks statistical control, and SAM and RP have low statistical efficiency. Genes expressed highly in two types of cell lines may hardly reach a large FC even if differences are rather large. Thus, these genes are unlikely to be identified as DE genes by FC and FC-based ranking methods (e.g., RP), and pathways related to drug-resistance may not be recognised. The converse of this statement is also true: genes expressed at low levels in two types of cell lines easily reach a large FC because of the low signal-to-noise ratio and large measurement errors^1^,^7^,^32^, which increases the risk of false positives. Due to normalisation and logarithmic transformation in SAM, the differences between the log-scaled expression levels of genes in two cell types are the logarithms of their FC ratios^5^, and therefore SAM shares the similar disadvantage with FC.

The reproducibility-based PD proposed in this paper can detect certain statistically significant, reproducible DE genes between two cell types and has an advantage over other more traditional methods in that it can identify genes with high expression and large differences but not a large FC. The results for four small-scale datasets for cancer cell line demonstrated that the DE genes identified by the reproducibility-based PD were significantly enriched in many biological pathways related to resistance to the corresponding drugs with a stringent FDR (<5%). However, many of these pathways were missed by FC, SAM and RP. The functional enrichment model indicates that the DE gene list can recognise enriched biological pathways only when there contains sufficient real DE genes^12^,^13^. Therefore, the functional enrichment analysis can be used to validate the reliability of the DE genes identified by the reproducibility-based PD.

By contrast, some pathways associated with drug resistance involve genes expressed at low levels. These pathways may be missed by the reproducibility-based PD. Therefore, we transformed the FC into a reproducibility-based PFC to detect DE genes expressed at low levels in small-scale cancer cell line datasets. With a stringent FDR (<5%), DE genes detected by the reproducibility-based PFC were enriched in many biological pathways, some of which were detected only by the reproducibility-based PFC. An example of such a pathway was the terpenoid backbone biosynthesis pathway in the MDA-MB-231 dataset, which is a core synthetic route for paclitaxel^30^,^31^. A total of 12 DE genes involved in the pathway were detected by the reproducibility-based PFC, 7 DE genes were detected by the reproducibility-based PD, and 6 DE genes were shared by the reproducibility-based PFC and the reproducibility-based PD. The other 6 DE genes solely detected by the reproducibility-based PFC exhibited low average expression levels in drug-resistant and drug-sensitive cell lines (30.64–159.82 and 17.71–174.56, respectively). Thus, the reproducibility-based PFC could compensate for the deficiency of the reproducibility-based PD and detect some pathways involving genes with low expression levels. Therefore, we recommend combining the reproducibility-based PD with the reproducibility-based PFC to detect DE genes in small-scale cancer cell line datasets with two or three technical replicates. However, this algorithm may be unsuitable for analysing small datasets of tissue samples with large biological variation. To detect DE genes for tissue samples, the sizes of samples should be large enough and SAM, RP or other strict statistical methods are recommended.

In addition, we recommend performing a quality evaluation with a consistency evaluation to exclude samples without significantly reproducible signals. This procedure will help eliminate the influence of experimental error. Notably, it is theoretically possible that the selection of reproducible sample pairs may introduce an ascertainment bias which may inflate false positives. However, this risk should actually be very low. Given that there is no biological difference between technical replicates of a cell, paired technical replicates of two types of cell line should produce highly consistent results (here, DE genes) when the technical variation of the measurement platforms is not too large. In other words, the prior probability of significant consistency between every two lists of DE genes detected from every two independent sample pairs should be very high, as demonstrated by the results that the consistency scores of the top n (n = 300, 600,……3000) genes sorted from almost all two independent pairs were significantly higher than expected by chance (binomial test, all p < 2.2E-16)(Fig. 1–c).

Obviously, the two parameters of the reproducibility-based PD, the CT and the initial step, can affect the process of selecting DE genes. Nevertheless, the enrichment pathways were rather stable when the parameter settings of the reproducibility-based PD were altered. We usually advise a CT of 90% and an initial step of 300. In consideration of global alterations in gene expression between two conditions^25^,^26^, if the number of DE genes is too few (<1,000), the consistency threshold can be reduced (e.g., 85%); if the number of DE genes is too many (>3,000), the consistency threshold can be increased (e.g., 95%).

We are also aware that the reproducibility-based PD and PFC ranking methods still cannot obtain DE genes by controlling the false discovery rate. However, compared with the FC method commonly used for analysing small-scale cancer cell line experiments, the reproducibility-based methods can decrease the false discovery rate by excluding irreproducible discoveries because the real DE genes between two types of cell lines should be reproducibly observed in paired technical replicates of the cell lines although reproducibility cannot ensure true discovery^11^,^33^,^34^. Intuitively, the lower the rate of false positives, the higher the consistency score of DE genes identified between two independent sample pairs, and vice versa. Indeed, the relationship between the CT (consistency threshold) and the FDR level is complex and deserves further study.

## Methods

### Microarray data and pre-processing

Four microarray datasets for drug-resistant and drug-sensitive cancer cell lines were used: cisplatin-resistant (CP70) and sensitive ovarian cancer cell lines (A2780)^35^, paclitaxel-resistant (resistant MDA-MB-231) and sensitive ER- breast cancer cell lines (parental MDA-MB-231), tamoxifen-resistant (LCC2) and sensitive ER+ breast cancer cell lines (MCF7)^36^ and 5-FU-resistant (resistant HCT116) and sensitive colorectal cancer cell lines (parental HCT116)^37^, referred to as CP70/A2780, MDA-MB-231, LCC2/MCF7 and HCT116 in this study, respectively. The datasets were acquired from the Gene Expression Omnibus^38^ and ArrayExpress^39^ databases, as described in detail in Table 2.

The three datasets (CP70/A2780, MDA-MB-231 and HCT116) generated from the Affymetrix microarray platform were pre-processed using the robust microarray average (RMA) algorithm^40^,^41^, and the other dataset (LCC2/MCF7) generated from the Illumina microarray platform was log2-transformed and quantile normalised. Then, each probe-set ID was mapped to its Entrez gene ID with the corresponding custom CDF files. If multiple probesets were mapped to the same gene, the expression value for the gene was defined as the arithmetic mean of the values of the multiple probesets (on the log2 scale).

### Calculation of pairwise difference and pairwise fold change

For a given pair *j* consisting of one drug-resistant (type R) sample and one drug-sensitive (type S) sample, the non-log-transformed expression values of gene *i* in the type R sample and S sample were denoted as 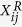 and 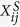, respectively. Then, the Pairwise Difference (*PD*_*ij*_) and Pairwise Fold Change (*PFC*_*ij*_) of each gene *i* between the two samples were calculated as follows, respectively:


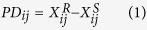



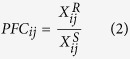


If the value of 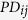
was larger (or smaller) than zero, then gene *i* was defined as up-regulated (or down-regulated) in type R samples. In the same case, if the value of 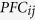
was larger (or smaller) than one, gene *i* was defined as up-regulated (or down-regulated) in type R samples.

### Reproducibility evaluation of two DE gene lists

For two DE gene lists sharing *k* DE genes, of which *s* genes had the same dysregulation directions (both up-regulated or down-regulated in the two DE gene lists) in type R samples, the consistency score was calculated as *s/k*. The probability of observing at least *s* of *k* DE genes with the same dysregulation directions by chance can be evaluated using the cumulative binomial distribution model as follows^42^:


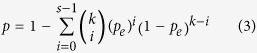


in which *p*_*e*_ is the probability of one gene having the same dysregulation direction in two DE gene lists by random chance (here, 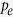 = 0.5). A DE gene list is considered significantly reproducible if the p-value of the consistency score < 0.01.

### Selection of reproducible independent sample pairs

Because there is no biological difference in technical replicates for a clonally derived cell line (type R or type S), we consider a sample pair including a technical replicate of type R and a technical replicate of type S as an experiment and every two experiments without any overlapping technical samples as independent sample pairs. We take the following steps to select the reproducible independent pairs. Suppose there are r_1_ technical replicates in type R cell and s_1_ technical replicates in type S cell, then they can form a total r_1_*s_1_ pairs of samples between type R and type S cells. (i) For each of the r_1_*s_1_ sample pairs, all genes are sorted in descending order according to the PD or PFC score rule;(ii) Then, the consistency of the dysregulation direction of the top n (e.g., n = 300) genes between every two independent sample pairs without overlapping samples is evaluated and the two independent sample pairs with the most significant *p*-value of the consistency score, denoted as R1-S1 and R2-S2, are selected. (iii)The remaining independent sample pairs (not including R1, R2, S1 and S2) are selected one by one if the consistency of the top *n* genes is significantly reproducible (*p*-value of the consistency score < 0.01) with the top *n* genes of at least one of the independent sample pairs selected previously. The above steps are repeated until no independent sample pairs can be determined.

### Selection of reproducible DE genes

After selecting reproducible independent sample pairs, we identify reproducible DE genes between every two independent sample pairs. For two independent sample pairs, pair A and pair B, all genes (N) are sorted in descending order according to the score rule (e.g., PD or PFC), denoted as list A and list B, respectively. Considering that genes with higher ranks in both list A and list B are more likely to be DE genes, we set an initial step length as *k* (e.g., 300 genes) and divide list A and list B into blocks with the size of *k* genes, respectively, and then identify genes in each of the decreasingly ranked blocks of list A that are significantly reproducible in the same top-ranked blocks of list B, denoted as degAB. The detail is as following:

(i) The consistency threshold (CT, e.g., 90%) and the initial step *k*(e.g., 300 genes) are set . List A and list B are then divided into blocks A(*i*) and B(*i*)(*i *= 1,2,…int(N*/ k*)) by *k*, for which A*(i)* and B*(i)* include the top genes ranked from ((*i*-1)*k +1) to i*k in list A and list B, respectively. (N*/k* is rounded down to yield int(N*/k*)).

(ii) For each A(i), beginning from A(1) and going down the decreasingly ranked blocks, the consistency of the dysregulation directions of overlapping genes between A*(i)* and blocks from B(1) to B(*i*) (denoted as B(1) ~ B*(i)*),are evaluated in sequence until the consistency score between A(m) and B(1) ~ B(m) is smaller than CT.

(iii) Then, the algorithm is moved forward one more block to evaluate the consistency of overlapping genes between A(m + 1)and B(1) ~ B(m +1) to avoid early termination of the search process which may occur when the number of overlapping genes between A(m) and B(1) ~ B(m) is too small to reach a stable consistency score. If the consistency of overlapping genes between A(m + 1)and B(1) ~ B(m + 1) is larger than CT, then return to (ii) and continue the algorithm, beginning from A(m + 1) and going down the decreasingly ranked blocks.

(iv) Otherwise, the algorithm turns to capture a part of genes with higher ranks in A(m) that may be significantly reproducible in list B before terminating, taking into account the possibility that the low consistency score between A(m) and B(1) ~ B(m) could be due to genes with lower ranks in A(m). The step *k* is shorten into *k*/2 and genes from A(m) or B (m) to the end of list A or list B are redivided by the new step *k*/2, respectively, and then return to (ii) to continue the algorithm, beginning from A’(m) (including the top-ranked int(*k/*2) genes in A(m)) and going down the decreasingly ranked blocks. This process is reiterated until no overlapping genes can be found. Then the algorithm is exited.

**Figure 4:**
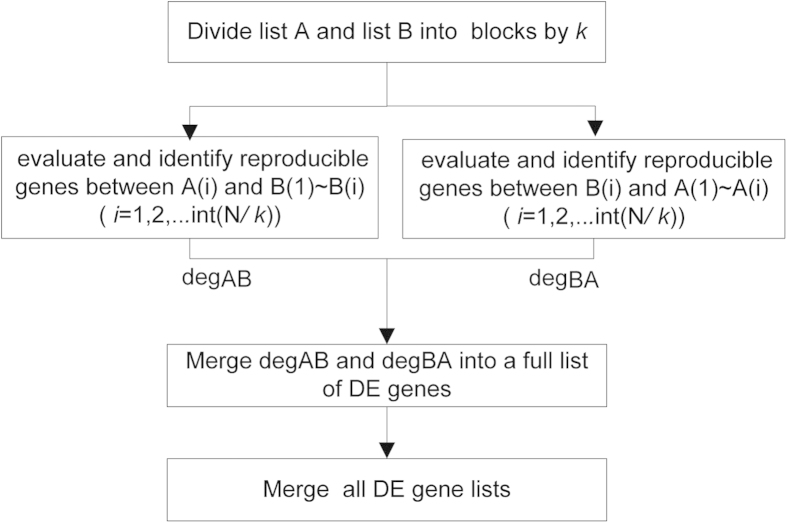
The flow chart of the reproducibility-based PD or PFC algorithm.

Then, the positions of list A and list B is exchanged, and the processes from (i) to (iv) are repeated to identify genes in each of the decreasingly ranked block of list B that are significantly reproducible in the same top-ranked blocks of list A, denoted as degBA. The two lists, degAB and degBA, are merged into a full list of significantly reproducible DE genes for sample pair A and sample pair B. The same process is done for every two independent sample pairs. Finally, all the identified DE gene lists are merged together after deleting genes with different dysregulation directions between any two DE genes lists. The flow chart of the algorithm is shown in Fig. 4 and the R-codes for the algorithm is available in Supplementary Methods.

### Pathway enrichment analysis

Functional enrichment analysis was performed based on the Kyoto Encyclopaedia of Genes and Genomes^43^. The hypergeometric distribution model was used to determine biological pathways that were significantly enriched with DE genes^44^, the probability of observing at least X genes in a pathway by chance can be computed as follow:


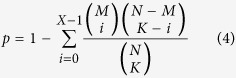


K is the number of DE genes identified from N genes in a dataset and X of them are annotated in a pathway with M genes.

The *p*-values were adjusted using the Benjamini and Hochberg procedure^45^, controlling the False Discovery Rate (FDR) at the 5% level.

## Additional Information

**How to cite this article**: Ao, L. *et al.* Identification of reproducible drug-resistance-related dysregulated genes in small-scale cancer cell line experiments. *Sci. Rep.* **5**, 11895; doi: 10.1038/srep11895 (2015).

## Supplementary Material

Supplementary Information

## Figures and Tables

**Table 1:** The reproducibility of the top 300 genes ranked by PD between every two independent pairs selected in the four datasets.

Dataset	Pair	Sample No.	Comparison	*K*	*S*	*S/K*(%)	*p*
CP70/A2780	pair 1	GSM709781 VS GSM709780	pair1–2	267	267	100.00%	<2.2E-16
	pair 2	GSM709782 VS GSM709778	pair1–3	151	142	94.04%	<2.2E-16
	pair 3	GSM709783 VS GSM709779	pair2–3	155	146	94.19%	<2.2E-16
MDA-MB-231	pair 1	GSM712688 VS GSM712682	pair1–2	200	194	97.00%	<2.2E-16
	pair 2	GSM712689 VS GSM712683	pair1–3	186	174	93.55%	<2.2E-16
	pair 3	GSM712690 VS GSM712684	pair2–3	179	167	93.30%	<2.2E-16
LCC2/MCF7	pair 1	GSM1326254 VS GSM1326258					
	pair 2	GSM1326255 VS GSM1326259	pair1–2	196	190	96.94%	<2.2E-16
HCT116	pair 1	MEXPl: 179508 VS MEXP: 179487					
	pair 2	MEXP: 179509 VS MEXP: 179486	pair1–2	147	136	92.52%	<2.2E-16

Note. pair1-2, pair1-3, pair2-3, the comparison of reproducibility between pair 1 and pair 2, pair1 and pair3, pair2 and pair3 in each dataset; *K*, the number of genes shared by two gene lists; *S*, the number of genes with same dysregulation directions; *S/K*(%), the consistency score; *p*, the probability of significant reproducibility.

**Table 2:** Details of the drug-resistant and drug-sensitive cancer cell line data used in this study.

Dataset Acc	Platform	Cell line	Phenotype	Drug	Size
GSE28646	GPL570	CP70/A2780	ovarian cancer	cisplatin	3 VS 3
GSE28784	GPL96	MDA-MB-231	ER− breast cancer	paclitaxel	3 VS 3
GSE54891	GPL10558	LCC2/MCF7	ER+ breast cancer	tamoxifen	2 VS 2
E-MEXP-390	GPL570	HCT116	colorectal cancer	5-FU	3 VS 3
