# Supplementary material for: Identification of reproducible drug-resistance-related dysregulated genes in small-scale cancer cell line experiments
Source: Sci Rep. 2015 Jul 15;5:11895. doi: 10.1038/srep11895 (PMC4502408; doi:10.1038/srep11895)
Supplement: Supplementary Information [file srep11895-s1.pdf]

Identification of reproducible drug-resistance-related dysregulated genes in small-scale cancer cell line experiments

Lu Ao, Haidan Yan, Tingting Zheng, Hongwei Wang, Mengsha Tong, Qingzhou Guan, Xiangyu Li, Hao Cai, Mengyao Li, Zheng Guo

Contents

1 Supplementary Figures
2 Supplementary Tables
3 Supplementary Methods

1 Supplementary Figures

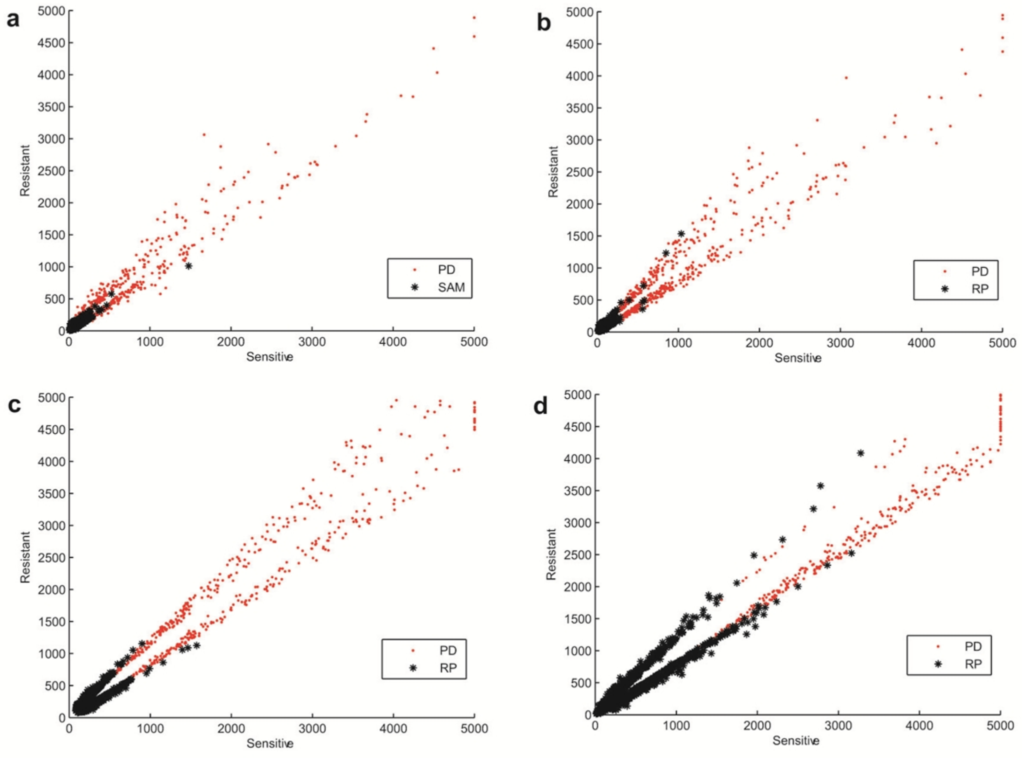

Supplementary Figure S1: **The distributions of DE genes exclusively detected by PD, SAM or RP**

DE genes exclusively detected by PD or SAM in the MDA-MB-231dataset (a), DE genes exclusively detected by PD or RP in the MDA-MB-231dataset (b), the LCC2/MCF7 dataset (c) and HCT116 datasets (d). The average expression levels of DE genes in resistant replicates and sensitive replicates were plotted. PD, SAM and RP represent the DE genes identified by PD, SAM and RP, respectively. The average expression level above 5,000 was set to 5,000.

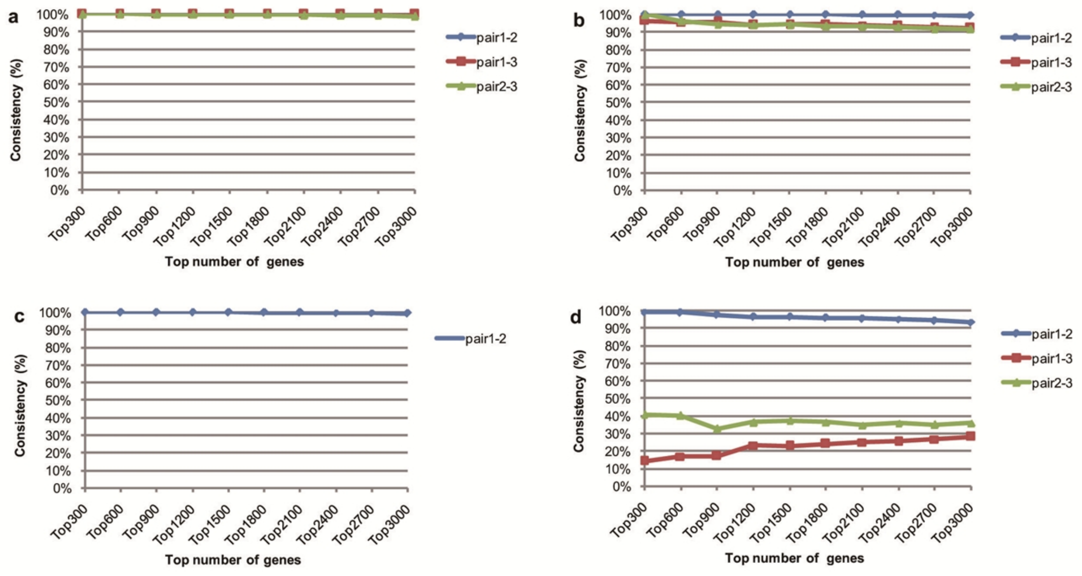

Supplementary Figure S2: **The evaluation of consistency scores between every two independent pairs**.

The consistency of top *n* (*n*=300, 600,...... 3000) genes ranked by PFC between every two independent pairs in the CP70/A2780 dataset (a), the MDA-MB-231dataset (b), the LCC2/MCF7 dataset (c) and the HCT116 dataset (d) . Consistency(%) ranges from 0%-100%, top 300 (top 600, etc.) are the top number of genes of two pairs ranked by PD. pair1-2, pair1-3, pair2-3 were the comparisons of reproducibility between pair 1 and pair 2, pair 1 and pair 3, pair 2 and pair 3 in each dataset, respectively.

2 Supplementary Tables

Supplementary Table S1: The enriched KEGG pathways of DE genes detected by PD in the CP70/A2780 dataset. $p(<0.05)$ was adjusted by Benjamini and Hochberg.

KEGG Pathway Name	*p*	Reference
CP70/A2780		
RNA transport	6.78E-09	
Protein processing in endoplasmic reticulum	7.53E-09	Mandic et al.[1], Rabik et al.[2]
Ribosome	2.74E-06	
Ubiquitin mediated proteolysis	7.18E-05	Gatti et al.[3], Motegi et al.[4]
p53 signaling pathway	7.73E-05	Shimodaira et al.[5], Lin et al.[6], Lin et al.[7]
Spliceosome	1.05E-05	
Cell cycle	1.12E-05	Stordal et al.[8], Basu et al.[9]
Aminoacyl-tRNA biosynthesis	2.98E-04	
Amino sugar and nucleotide sugar metabolism	1.36E-03	

Supplementary Table S2: The enriched KEGG pathways of DE genes detected by PD in the MDA-MB-231 dataset. $p(<0.05)$ was adjusted by Benjamini and Hochberg.

KEGG Pathway Name	*p*	Reference
MDA-MB-231		
Oxidative phosphorylation	5.67E-12	
Ribosome	4.48E-11	
Protein processing in endoplasmic reticulum	4.31E-05	Wu et al.[10]
Phagosome	5.62E-04	
Lysosome	5.64E-04	Broker et al.[11], Groth-Pederson et al.[12]
Spliceosome	5.64E-04	Bonnal et al.[13]
Aminoacyl-tRNA biosynthesis	8.45E-04	
Biosynthesis of amino acids	1.36E-03	
Pyruvate metabolism	1.72E-03	Lu et al.[14], Zhao et al.[15],
Antigen processing and presentation	2.26E-03	
Citrate cycle (TCA cycle)	2.27E-03	

Supplementary Table S3: The enriched KEGG pathways of DE genes detected by PD in the LCC2/MCF7 datasets. *p*(<0.05) was adjusted by Benjamini and Hochberg.

KEGG Pathway Name	*p*	Reference
LCC2/MCF7		
Oxidative phosphorylation	2.20E-16	Huber-Keener et al.[16]
Ribosome	2.20E-16	
Proteasome	2.10E-10	Huber-Keener et al.[16]
RNA transport	2.30E-07	
Spliceosome	6.42E-07	Bonnal et al.[13]
Pyrimidine metabolism	3.34E-06	
Glutathione metabolism	2.71E-05	Ryu et al.[17]
Protein processing in endoplasmic reticulum	5.34E-05	Wu et al.[10]
Carbon metabolism	6.19E-05	Brown et al.[18]
Cell cycle	7.16E-05	Musgrove et al.[19], Riggins et al.[20]
RNA degradation	2.82E-05	
Lysosome	1.13E-04	Nagelkerke et al.[21], Groth-Pedersen et al.[12]
Biosynthesis of amino acids	4.84E-04	
Citrate cycle (TCA cycle)	5.18E-04	
Valine, leucine and isoleucine degradation	1.25E-03	
Aminoacyl-tRNA biosynthesis	1.25E-03	
Cysteine and methionine metabolism	1.60E-03	Ryu et al.[17]
RNA polymerase	2.73E-03	
Ribosome biogenesis in eukaryotes	3.84E-03	

Supplementary Table S4: The enriched KEGG pathways of DE genes detected by PD in the HCT116 datasets. *p*(<0.05) was adjusted by Benjamini and Hochberg.

KEGG Pathway Name	*p*	Reference
HCT116		
Oxidative phosphorylation	2.20E-16	
Ribosome	2.20E-16	
Proteasome	7.22E-10	
RNA transport	9.79E-09	
Spliceosome	6.67E-08	
DNA replication	1.22E-07	Martino-Echarri et al.[22], Longley et al.[23]
Nucleotide excision repair	5.20E-05	
Ribosome biogenesis in eukaryotes	5.25E-05	
Mismatch repair	5.29E-05	Vymetalkova et al.[24], Longley et al.[23], Meyers et al.[25]
Protein export	8.02E-05	
Cardiac muscle contraction	2.54E-04	
Base excision repair	1.70E-03	Meyers et al.[25], Wyatt et al.[26]

Supplementary Table S5: The enriched GO pathways of DE genes detected by PD in the CP70/A2780 datasets. *p*(<0.05) was adjusted by Benjamini and Hochberg.

GO Pathway Name	*p*
CP70/A2780	
microtubule cytoskeleton organization	2.20E-16
mitotic prometaphase	2.20E-16
purine nucleotide catabolic process	2.20E-16
transcription termination, DNA-dependent	1.22E-15
transcription from RNA polymerase II promoter	5.25E-14
RNA processing	4.90E-13
tRNA metabolic process	4.57E-12
cellular amino acid metabolic process	2.41E-10
vesicle targeting	3.68E-10
apoptosis	2.57E-08
response to DNA damage stimulus	3.78E-08
mitochondrion organization	4.19E-08
mitosis	4.68E-08
ribonucleotide catabolic process	5.84E-08
negative regulation of macromolecule metabolic process	5.84E-08
posttranscriptional regulation of gene expression	9.33E-08
microtubule-based transport	1.70E-07
programmed cell death	1.93E-07
viral reproduction	2.08E-07
mRNA metabolic process	2.75E-07
viral transcription	3.43E-07
endocrine pancreas development	1.28E-06
negative regulation of cellular metabolic process	1.86E-06
negative regulation of type I interferon production	3.19E-06
regulation of microtubule-based process	3.26E-06
cellular response to stress	3.75E-06
cellular protein localization	3.83E-06
cellular macromolecular complex assembly	4.53E-06
cellular macromolecule biosynthetic process	6.05E-06
response to endoplasmic reticulum stress	1.03E-05
ribosome biogenesis	2.21E-05
negative regulation of apoptosis	2.41E-05
macromolecule modification	4.05E-05
cellular macromolecule catabolic process	4.69E-05
cellular protein metabolic process	4.72E-05
sulfur compound biosynthetic process	1.16E-04
interspecies interaction between organisms	1.53E-04
establishment of protein localization	1.54E-04
purine ribonucleoside metabolic process	1.64E-04
Golgi vesicle transport	2.13E-04
organelle fusion	2.17E-04
positive regulation of cellular process	2.70E-04
mRNA transport	2.92E-04
nuclear transport	4.28E-04
cofactor biosynthetic process	4.83E-04

chromosome organization	5.46E-04
cell division	5.89E-04
interphase of mitotic cell cycle	5.89E-04
establishment of organelle localization	6.58E-04
regulation of cell cycle	7.50E-04
membrane organization	8.69E-04
organelle assembly	8.85E-04
protein complex subunit organization	8.92E-04
ribonucleoprotein complex subunit organization	9.69E-04
cellular component disassembly at cellular level	1.05E-03

Supplementary Table S6: The enriched GO pathways of DE genes detected by PD in the MDA-MB-231 datasets. *p*(<0.05) was adjusted by Benjamini and Hochberg.

GO Pathway Name	*p*
MDA-MB-231	
DNA repair	2.20E-16
RNA processing	1.52E-13
tRNA metabolic process	3.16E-12
translation	3.75E-12
translational initiation	1.98E-11
translational elongation	2.03E-11
tRNA aminoacylation for protein translation	5.89E-11
protein folding	1.07E-10
membrane protein ectodomain proteolysis	8.79E-10
cellular amino acid metabolic process	2.10E-09
regulation of cellular amino acid metabolic process	8.31E-09
coenzyme metabolic process	1.58E-08
vesicle targeting	2.48E-08
DNA damage response, signal transduction by p53 class mediator resulting in cell cycle arrest	6.21E-08
response to oxidative stress	7.07E-08
mitochondrial membrane organization	1.78E-07
macromolecule catabolic process	2.32E-07
aerobic respiration	5.09E-07
oligosaccharide metabolic process	7.46E-07
regulation of cell death	1.19E-06
programmed cell death	2.08E-06
protein transport	5.10E-06
ATP hydrolysis coupled proton transport	6.06E-06
viral reproduction	1.19E-05
cellular membrane organization	1.50E-05
mRNA metabolic process	1.66E-05
protein N-linked glycosylation via asparagine	2.08E-05
antigen processing and presentation	3.59E-05
viral reproductive process	3.70E-05
respiratory electron transport chain	4.30E-05
endocrine pancreas development	5.97E-05
negative regulation of cellular protein metabolic process	6.95E-05
ncRNA processing	8.17E-05
cellular macromolecular complex subunit organization	9.47E-05
ribosome biogenesis	1.07E-04
protein complex disassembly	1.08E-04
post-translational protein modification	1.63E-04
cellular catabolic process	1.97E-04
cellular carbohydrate metabolic process	1.98E-04
interspecies interaction between organisms	2.07E-04
cell redox homeostasis	2.39E-04
intracellular transport	2.42E-04
de novo posttranslational protein folding	2.62E-04
negative regulation of ubiquitin-protein ligase activity	2.69E-04

involved in mitotic cell cycle	
positive regulation of ubiquitin-protein ligase activity involved in mitotic cell cycle	3.92E-04
cellular macromolecule localization	5.50E-04
protein complex subunit organization	5.99E-04
cellular component disassembly at cellular level	7.03E-04

Supplementary Table S7: The enriched GO pathways of DE genes detected by PD in the LCC2/MCF7 datasets. *p*(<0.05) was adjusted by Benjamini and Hochberg.

GO Pathway Name	*p*
LCC2/MCF7	
G1/S transition of mitotic cell cycle	2.20E-16
S phase of mitotic cell cycle	2.20E-16
protein polyubiquitination	2.20E-16
M/G1 transition of mitotic cell cycle	2.20E-16
RNA splicing, via transesterification reactions	2.20E-16
cytokinesis	2.20E-16
antigen processing and presentation of peptide antigen via MHC class I	2.20E-16
generation of precursor metabolites and energy	2.20E-16
tricarboxylic acid cycle	2.20E-16
ATP catabolic process	2.45E-14
DNA metabolic process	8.82E-14
tRNA metabolic process	9.17E-14
translation	9.44E-14
protein folding	1.20E-13
cellular amino acid metabolic process	3.24E-13
regulation of cellular amino acid metabolic process	3.96E-13
coenzyme metabolic process	8.47E-13
DNA damage response, signal transduction by p53 class mediator resulting in cell cycle arrest	9.66E-13
mitochondrion organization	1.82E-12
mitotic cell cycle checkpoint	2.24E-12
regulation of mitotic cell cycle	2.25E-12
cullin deneddylation	2.37E-12
negative regulation of macromolecule metabolic process	5.70E-12
protein transport	7.00E-12
ATP hydrolysis coupled proton transport	1.79E-11
viral reproduction	2.02E-11
mRNA metabolic process	1.07E-10
rRNA metabolic process	2.19E-10
viral transcription	2.32E-10
cellular component disassembly	4.24E-10
viral reproductive process	2.14E-08
respiratory electron transport chain	5.90E-08
endocrine pancreas development	9.56E-08
anaphase-promoting complex-dependent proteasomal ubiquitin-dependent protein catabolic process	1.96E-07
negative regulation of cellular metabolic process	2.12E-07
protein modification by small protein conjugation	9.21E-07
ncRNA processing	1.05E-06
cellular macromolecular complex subunit organization	1.06E-06
response to endoplasmic reticulum stress	1.10E-06
response to topologically incorrect protein	2.57E-06
ribosome biogenesis	4.71E-06
regulation of apoptosis	5.92E-06

proteasomal ubiquitin-dependent protein catabolic process	6.88E-06
dicarboxylic acid metabolic process	1.08E-05
cellular catabolic process	1.14E-05
cellular macromolecule catabolic process	1.36E-05
small molecule biosynthetic process	2.03E-05
interspecies interaction between organisms	2.57E-05
intracellular transport	2.64E-05
organelle fission	2.95E-05
RNA transport	3.23E-05
de novo posttranslational protein folding	3.38E-05
cofactor biosynthetic process	4.13E-05
chromosome organization	4.78E-05
cell division	7.90E-05
interphase of mitotic cell cycle	1.29E-04
negative regulation of ubiquitin-protein ligase activity involved in mitotic cell cycle	1.43E-04
positive regulation of ubiquitin-protein ligase activity	1.65E-04
establishment of organelle localization	3.30E-04
oxidation-reduction process	4.35E-04
macromolecular complex assembly	5.95E-04
protein complex subunit organization	7.05E-04
ribonucleoprotein complex subunit organization	9.43E-04

Supplementary Table S8: The enriched GO pathways of DE genes detected by PD in the HCT116 datasets. *p*(<0.05) was adjusted by Benjamini and Hochberg.

GO Pathway Name	*p*
HCT116	
mitotic sister chromatid segregation	2.20E-16
cell cycle checkpoint	2.20E-16
G1/S transition of mitotic cell cycle	2.20E-16
S phase of mitotic cell cycle	2.20E-16
protein polyubiquitination	2.20E-16
M/G1 transition of mitotic cell cycle	2.20E-16
mitotic prometaphase	2.20E-16
M phase	2.20E-16
RNA splicing, via transesterification reactions	2.20E-16
telomere maintenance via recombination	4.44E-16
antigen processing and presentation of peptide antigen via MHC class I	6.66E-15
generation of precursor metabolites and energy	1.22E-14
oxidative phosphorylation	4.72E-14
ATP catabolic process	4.87E-14
DNA replication	3.81E-12
DNA-dependent DNA replication	9.01E-12
DNA-dependent DNA replication initiation	1.70E-11
DNA strand elongation involved in DNA replication	1.97E-11
DNA repair	2.21E-11
transcription-coupled nucleotide-excision repair	3.20E-11
nucleotide-excision repair, DNA gap filling	7.32E-11
transcription elongation from RNA polymerase II promoter	2.39E-10
termination of RNA polymerase II transcription	2.76E-10
RNA export from nucleus	4.18E-10
translation	5.04E-10
translational initiation	9.23E-10
translational elongation	1.64E-09
translational termination	3.66E-09
protein folding	4.00E-09
cellular amino acid metabolic process	4.07E-09
regulation of cellular amino acid metabolic process	1.90E-08
mitochondrial transport	2.39E-08
DNA damage response, signal transduction by p53 class mediator resulting in cell cycle arrest	3.97E-08
mitochondrion organization	4.38E-08
chromosome segregation	6.24E-08
mitotic cell cycle checkpoint	4.11E-07
RNA splicing	1.11E-06
aerobic respiration	1.23E-06
RNA modification	1.23E-06
cullin deneddylation	3.39E-06
protein transport	5.64E-06
ATP synthesis coupled proton transport	1.42E-05
viral reproduction	1.68E-05

mRNA metabolic process	2.13E-05
viral transcription	2.47E-05
respiratory electron transport chain	2.77E-05
endocrine pancreas development	5.15E-05
anaphase-promoting complex-dependent proteasomal ubiquitin-dependent protein catabolic process	6.82E-05
positive regulation of protein ubiquitination	7.15E-05
telomere maintenance via semi-conservative replication	7.19E-05
cellular macromolecular complex assembly	7.82E-05
ncRNA metabolic process	9.82E-05
ribosome biogenesis	1.24E-04
proteasomal ubiquitin-dependent protein catabolic process	1.47E-04
cellular macromolecule catabolic process	1.56E-04
interspecies interaction between organisms	1.86E-04
intracellular transport	2.27E-04
mRNA transport	2.48E-04
de novo posttranslational protein folding	2.58E-04
protein homotetramerization	3.47E-04
cell division	4.21E-04
negative regulation of ubiquitin-protein ligase activity involved in mitotic cell cycle	4.98E-04
positive regulation of ubiquitin-protein ligase activity involved in mitotic cell cycle	9.64E-04

Supplementary Table S9: The reproducibility of the top 300 genes ranked by PFC between every two independent pairs selected in the four datasets.

Dataset	Pair	Sample-pairs	Comparison	*K*	*S*	*S/K*(%)	*p*
A2780	pair 1	GSM709781 VS GSM709779	pair1-2	215	215	100.00%	<2.2E-16
	pair 2	GSM709782 VS GSM709778	pair1-3	269	269	100.00%	<2.2E-16
	pair 3	GSM709783 VS GSM709780	pair2-3	221	221	100.00%	<2.2E-16
MDA-MB-231	pair 1	GSM712688 VS GSM712684	pair1-2	169	169	97.00%	<2.2E-16
	pair 2	GSM712689 VS GSM712683	pair1-3	133	128	96.24%	<2.2E-16
	pair 3	GSM712690 VS GSM712682	pair2-3	122	122	100.00%	<2.2E-16
LCC2/MCF7	pair 1	GSM1326254 VS GSM1326258	pair1-2	202	202	100.00%	<2.2E-16
	pair 2	GSM1326255 VS GSM1326259					
HCT116	pair 1	MEXP:179508 VS MEXP:179487	pair1-2	106	105	99.06%	<2.2E-16
	pair 2	MEXP:179509 VS MEXP:179486					

Supplementary Table S10: The enriched pathways of DE genes detected by PFC in the CP70/A2780, MDA-MB-231, LCC2/MCF7 and HCT116 datasets. *p*(<0.05) was adjusted by Benjamini and Hochberg.

KEGG Pathway Name	*p*	Reference
CP70/A2780		
Protein processing in endoplasmic reticulum	7.27E-05	Mandic et al.[1], Rabik et al.[2]
p53 signaling pathway	1.68E-04	Shimodaira et al.[5], Lin et al.[6], Lin et al.[7]
MDA-MB-231		
Lysosome	1.32E-06	Broker et al.[11], Groth-Pederson et al.[12]
Pyruvate metabolism	8.78E-04	Lu et al.[14], Zhao et al.[15]
Citrate cycle (TCA cycle)	6.83E-04	
Terpenoid backbone biosynthesis	8.11E-04	Boghigian et al.[27], Hao et al.[28]
LCC2/MCF7		
Oxidative phosphorylation	1.64E-06	Huber-Keener et al.[16]
Ribosome	5.10E-05	
Proteasome	5.75E-06	Huber-Keener et al.[16]
Pyrimidine metabolism	5.36E-05	
Cell cycle	1.78E-04	Musgrove et al.[19], Riggins et al.[20]
Lysosome	3.51E-06	Nagelkerke et al.[21], Groth-Pedersen et al.[12]
Vitamin B6 metabolism	9.62E-04	
HCT116		
DNA replication	4.23E-06	Martino-Echarri et al.[22], Longley et al.[23]

Reference

1 Mandic, A., Hansson, J., Linder, S. & Shoshan, M. C. Cisplatin induces endoplasmic reticulum stress and nucleus-independent apoptotic signaling. *J Biol Chem* **278**, 9100-9106, doi:10.1074/jbc.M210284200 (2003).

2 Rabik, C. A. & Dolan, M. E. Molecular mechanisms of resistance and toxicity associated with platinating agents. *Cancer Treat Rev* **33**, 9-23, doi:10.1016/j.ctrv.2006.09.006 (2007).

3 Gatti, L. *et al.* Ubiquitin-proteasome genes as targets for modulation of cisplatin sensitivity in fission yeast. *BMC Genomics* **12**, 44, doi:10.1186/1471-2164-12-44 (2011).

4 Motegi, A., Murakawa, Y. & Takeda, S. The vital link between the ubiquitin-proteasome pathway and DNA repair: impact on cancer therapy. *Cancer Lett* **283**, 1-9, doi:10.1016/j.canlet.2008.12.030 (2009).

5 Shimodaira, H., Yoshioka-Yamashita, A., Kolodner, R. D. & Wang, J. Y. Interaction of mismatch repair protein PMS2 and the p53-related transcription factor p73 in apoptosis response to cisplatin. *Proc Natl Acad Sci U S A* **100**, 2420-2425, doi:10.1073/pnas.0438031100 (2003).

6 Lin, X. *et al.* P53 modulates the effect of loss of DNA mismatch repair on the sensitivity of human colon cancer cells to the cytotoxic and mutagenic effects of cisplatin. *Cancer Res* **61**, 1508-1516 (2001).

7 Lin, X. & Howell, S. B. DNA mismatch repair and p53 function are major determinants of the rate of development of cisplatin resistance. *Mol Cancer Ther* **5**, 1239-1247, doi:10.1158/1535-7163.MCT-05-0491 (2006).

8 Stordal, B. & Davey, M. Understanding cisplatin resistance using cellular models. *IUBMB Life* **59**, 696-699, doi:10.1080/15216540701636287 (2007).

9 Basu, A. & Krishnamurthy, S. Cellular responses to Cisplatin-induced DNA damage. *J Nucleic Acids* **2010**, doi:10.4061/2010/201367 (2010).

10 Wu, Y., Fabritius, M. & Ip, C. Chemotherapeutic sensitization by endoplasmic reticulum stress: increasing the efficacy of taxane against prostate cancer. *Cancer Biol Ther* **8**, 146-152 (2009).

11 Broker, L. E. *et al.* Cathepsin B mediates caspase-independent cell death induced by microtubule stabilizing agents in non-small cell lung cancer cells. *Cancer Res* **64**, 27-30 (2004).

12 Groth-Pedersen, L. & Jaattela, M. Combating apoptosis and multidrug resistant cancers by targeting lysosomes. *Cancer Lett* **332**, 265-274, doi:10.1016/j.canlet.2010.05.021 (2013).

13 Bonnal, S., Vigevani, L. & Valcarcel, J. The spliceosome as a target of novel antitumour drugs. *Nat Rev Drug Discov* **11**, 847-859, doi:10.1038/nrd3823 (2012).

14 Lu, C. W. *et al.* Overexpression of pyruvate dehydrogenase kinase 3 increases drug resistance and early recurrence in colon cancer. *Am J Pathol* **179**, 1405-1414, doi:10.1016/j.ajpath.2011.05.050 (2011).

15 Zhao, Y., Butler, E. B. & Tan, M. Targeting cellular metabolism to improve cancer therapeutics. *Cell Death Dis* **4**, e532, doi:10.1038/cddis.2013.60 (2013).

16 Huber-Keener, K. J. *et al.* Differential gene expression in tamoxifen-resistant breast cancer cells revealed by a new analytical model of RNA-Seq data. *PLoS One* **7**, e41333, doi:10.1371/journal.pone.0041333 (2012).

17 Ryu, C. S. *et al.* Elevation of cysteine consumption in tamoxifen-resistant MCF-7 cells. *Biochem Pharmacol* **85**, 197-206, doi:10.1016/j.bcp.2012.10.021 (2013).

18 Browne, B. C. *et al.* Global characterization of signalling networks associated with tamoxifen resistance in breast cancer. *FEBS J* **280**, 5237-5257, doi:10.1111/febs.12441 (2013).
19 Musgrove, E. A. & Sutherland, R. L. Biological determinants of endocrine resistance in breast cancer. *Nat Rev Cancer* **9**, 631-643, doi:10.1038/nrc2713 (2009).
20 Riggins, R. B., Schrecengost, R. S., Guerrero, M. S. & Bouton, A. H. Pathways to tamoxifen resistance. *Cancer Lett* **256**, 1-24, doi:10.1016/j.canlet.2007.03.016 (2007).
21 Nagelkerke, A. *et al.* LAMP3 is involved in tamoxifen resistance in breast cancer cells through the modulation of autophagy. *Endocr Relat Cancer* **21**, 101-112, doi:10.1530/ERC-13-0183 (2014).
22 Martino-Echarri, E., Henderson, B. R. & Brocardo, M. G. Targeting the DNA replication checkpoint by pharmacologic inhibition of Chk1 kinase: a strategy to sensitize APC mutant colon cancer cells to 5-fluorouracil chemotherapy. *Oncotarget* **5**, 9889-9900 (2014).
23 Longley, D. B., Harkin, D. P. & Johnston, P. G. 5-fluorouracil: mechanisms of action and clinical strategies. *Nat Rev Cancer* **3**, 330-338, doi:10.1038/nrc1074 (2003).
24 Vymetalkova, V. *et al.* Variations in mismatch repair genes and colorectal cancer risk and clinical outcome. *Mutagenesis* **29**, 259-265, doi:10.1093/mutage/geu014 (2014).
25 Meyers, M. *et al.* DNA mismatch repair-dependent response to fluoropyrimidine-generated damage. *J Biol Chem* **280**, 5516-5526, doi:10.1074/jbc.M412105200 (2005).
26 Wyatt, M. D. & Wilson, D. M., 3rd. Participation of DNA repair in the response to 5-fluorouracil. *Cell Mol Life Sci* **66**, 788-799, doi:10.1007/s00018-008-8557-5 (2009).
27 Boghigian, B. A., Myint, M., Wu, J. & Pfeifer, B. A. Simultaneous production and partitioning of heterologous polyketide and isoprenoid natural products in an Escherichia coli two-phase bioprocess. *J Ind Microbiol Biotechnol* **38**, 1809-1820, doi:10.1007/s10295-011-0969-9 (2011).
28 Hao da, C., Ge, G., Xiao, P., Zhang, Y. & Yang, L. The first insight into the tissue specific taxus transcriptome via Illumina second generation sequencing. *PLoS One* **6**, e21220, doi:10.1371/journal.pone.0021220 (2011).

3 Supplementary Methods

The R-code for the PD or PFC algorithm based on reproducibility evaluation

The following is a specific example of using the algorithm to identify reproducible DE genes in small-scale cell line dataset.

For a gene expression matrix with N genes (rows) and M samples (columns), there are m_1 samples in type R and m_2 samples in type. The expression values are non-log-transformed.

full_deg	The reproducibility evaluation in small-scale cell line dataset

Description

identify reproducible DE genes in small-scale cell line dataset

Usage

full_deg (expR,expS,geneid,step1,top_num,pval,consist_th,method)

Arguments

expR	a (non-empty) numeric matrix(or table) of data values of m_1 samples in type R
expS	a (non-empty) numeric matrix(or table) of data values of m_2 samples in type S
geneid	a (non-empty) numeric vector of Entrez gene IDs. The length of *geneid* must be the same as the number of rows in the *expR* and *expS* Value
step1	the initial step
top_num	the number of top genes to evaluate the consistency of dysregulation direction between every two pairs
pval	the statistic control of reproducibility
consist_th	the consistency threshold
method	'PD' or 'PFC'

Value

index	all possible combinations of pairs between type R and type S
all_pairs	the reproducible pairs
select_pair	the reproducible independent pairs selected by reproducibility evaluation
select_deg	all genes in every *select_pair* sorted by ‘method’
full	DE genes identified by the algorithm of reproducibility evaluation

Examples

x<-full_deg(expR,expS,geneid,300,300,0.01,0.9,'AD')

```
##compute the PD values of all genes in one sample pair
pair_diff<-function(expR,expS,geneid){
 diff<- expR-expS
 diff<-cbind(geneid,diff)
 diff1<-diff[diff[,2]>0,]
 diff1<-cbind(diff1,rep(1,nrow(diff1)))
 diff2<-diff[diff[,2]<0,]
 diff2<-cbind(abs(diff2),rep(0,nrow(diff2)))
 diff<-matrix(rbind(diff1,diff2),ncol=3)
 pair_diff<-diff[order(diff[,2],decreasing=T),]
return(pair_diff)
}

## compute the PFC values of all genes in one sample pair
pair_fchange<-function(expR,expS,geneid){
 fchange1<- expR/expS
 fchange1<-cbind(geneid,fchange1)
 fchange1<-fchange1[fchange1[,2]>1,]
 fchange1<-cbind(fchange1,rep(1,nrow(fchange1)))
 fchange2<-expS/expR
 fchange2<-cbind(geneid,fchange2)
 fchange2<-fchange2[fchange2[,2]>1,]
 fchange2<-cbind(fchange2,rep(0,nrow(fchange2)))
 fchange<-matrix(rbind(fchange1,fchange2),ncol=3)
 pair_fchange<-fchange[order(fchange[,2],decreasing=T),]
return(pair_fchange)
}

## # evaluate the reproducibility of  consistency score between two gene lists
inter_com_gene<-function(deg1,deg2){
 deg1=as.matrix(deg1)
 deg2=as.matrix(deg2)
 if (ncol(deg1)==1){ deg1=t(deg1)}
 com<-intersect(deg1[,1],deg2[,1])
 if (length(com )==0){
  return (list (sel<-0,com<-0 ,consist_ratio=sel/com))
  }
  else{
 com1<-match(com,deg1[,1])
 common1<-deg1[com1,]
 com2<-match(com,deg2[,1])
 common2<-deg2[com2,]
 common1=as.matrix(common1)
```

```
 common2=as.matrix(common2)
 if (ncol(common1)==1){
   common1=t(common1)
   common2=t(common2)
}
 pair<-(common1[,3]==1&common2[,3]==1|common1[,3]==0&common2[,3]==0)
 sel<-common1[pair==TRUE,]
 sel=as.matrix(sel)
if (nrow(sel)==0){
  return(list(sel<-0,com=com,consist_ratio=0))}
else{ if(ncol(sel)==1)
{
  sel=t(sel)
}

 p1<-binom.test(nrow(sel)-1,length(com),p=0.5,alternative='greater')
 consist_ratio<-nrow(sel)/length(com)
return(list(sel=sel,com=com,consist_ratio=consist_ratio,p1=p1))}


## # select the reproducible independent sample pairs
pair_select<-function(expR,expS,geneid,top_num,pval,method){
 n1<-ncol(expR)
 n2<-ncol(expS)
 m1<-nrow(expR)
index<-NULL
k<-c(0)
deg<-NULL
for(i in 1:n1){         #compute the PD or PFC value in all pairs
   for(j in 1:n2){
    k=k+1
  if ('PD'%in%method){
  pairdiff<-pair_diff(expR[,i],expS[,j],geneid)
  deg[[k]]<-pairdiff  }
  if('PFC'%in%method){
  pairfchange<-pair_fchange(expR[,i],expS[,j],geneid)
  deg[[k]]<-pairfchange }
 index<-rbind(index,c(i,j))
}
}
all_pairs<-NULL
for(i in 1:(k-1)){      #select the pairs which are reproducible from all pairs
   for(j in (i+1):k){
   a=deg[[i]]
```

```
  b=deg[[j]]
  com_gene<-inter_com_gene(a[1:top_num,],b[1:top_num,])
 if(is.null(com_gene$p1$p.value)){ next
}
  else{if(com_gene$p1$p.value<pval) all_pairs<-
rbind(all_pairs,c(i,j,index[i,],index[j,],com_gene$consist_ratio,com_gene$p1$p.value
))}
}
}
 all_pairs<-all_pairs[order(all_pairs[,8],-all_pairs[,7]),]
m2<-nrow(all_pairs)
select_pair<-NULL
R=NULL
S=NULL
for(i in 1:m2){                    # the sample have been included in R or in S  are excluded
  loc1<-(all_pairs[i,c(3,5)]) %in% R
  loc2<-(all_pairs[i,c(4,6)]) %in% S
if(loc1[1]==FALSE&loc1[2]==FALSE&loc2[1]==FALSE&loc2[2]==FALSE&all_p
airs[i,3]!=all_pairs[i,5]&all_pairs[i,4]!=all_pairs[i,6]){
  select_pair<-cbind(select_pair,all_pairs[i,1],all_pairs[i,2])
  R<-index[select_pair[1,],1]
  S<-index[select_pair[1,],2]
  break
 }
}
fdr<-pval
for (i in 1:k)
{
  count<-0
  loc1<-(index[i,1]) %in% R
  loc2<-(index[i,2]) %in% S
  if(loc1[1]==FALSE&loc2[1]==FALSE)
 {
   for(j in 1:m2)
    {
       if(all_pairs[j,1]==i&all_pairs[j,5]!=index[i,1]&all_pairs[j,6]!=index[i,2])
        {
           if(all_pairs[j,8]<fdr) count=count+1
        }
      if(all_pairs[j,2]==i&all_pairs[j,3]!=index[i,1]&all_pairs[j,4]!=index[i,2])
        {
           if(all_pairs[j,8]<fdr) count=count+1
        }
    }
```

```
  }
if (count>0)
    {
     select_pair<-cbind(select_pair,c(i))
    # R<-cbind(R,c(index[i,1]))
     R<-c(R,index[i,1])
     #S<-cbind(S,c(index[i,2]))
     S<-c(S,index[i,2])
    }
}
  select_deg<-NULL
 for (i in 1:length(select_pair)){
   select_deg[[i]]<-deg[[select_pair[i]]]
 }
return(list(index=index,all_pairs=all_pairs,select_pair=select_pair,select_deg=select_
deg))
}


## #select the reproducible DE genes from deg1 to deg2
deg_select<-function(deg1,deg2,step1,consist_th)
{
  count<-0
  flag<-0
  com_num<-NULL
  sel_num<-NULL
  consist_ratio<-NULL
  sel<-NULL
  min_len=min(nrow(deg1),nrow(deg2))
  step<-NULL
  n<-min_len%/%step1
  j<-0
  while (step1>0)
   {
     a<-sum(step)
     n<-(min_len-a)%/%step1
     for(i in 1:n)
         {
         deg_a<-deg1[(a+(i-1)*step1+1):(a+i*step1),]
         deg_b<-deg2[1:(a+i*step1),]
         com<-inter_com_gene(deg_a,deg_b)
         sel[[j+i]]<-com$sel
                      com_num <-c(com_num,length(com$com) )
                      sel_num<-c(sel_num,length(com$sel)/3)
```

```
        consist_ratio<-c(consist_ratio , com$consist_ratio)
                    if (is.nan(com$consist_ratio)|com$consist_ratio==0)     #no overlap
                        { flag<-1
                         break  }
        else {
             flag<-0
             if (com$consist_ratio<consist_th) count <-count +1 else count <-0
                        if (count==2) break
                 }
          if (count==2){        #go backward two blocks
            j<-j+i-2
                  sel[[j+2]]=NULL
                  sel[[j+1]]=NULL
                  com_num<-com_num[-c(j+1,j+2)]
                  sel_num <-sel_num [-c(j+1,j+2)]
                  consist_ratio<-consist_ratio[-c(j+1,j+2)]
        step<-rbind(step,matrix(rep(step1,i-2)))
        step2<-step1
        step1<-step1 %/%2
        count <-0}
          else { if (flag ==1){
                  sel[[j+i]]=NULL
                  com_num <-com_num[-c(j+i)]
                  sel_num <-sel_num[-c(j+i)]
                  consist_ratio<-consist_ratio[-c(j+i)]
        step <-  rbind(step ,matrix(rep(step1,i-1)))
        break
        }
                  }
  }
  L=length(sel)
  sel_last<-NULL
  for (k in 1:L){
    sel_last<-rbind (sel_last,sel [[k]])
         }
   if (length(which(is.na(sel_last)))==L) {
       print("no DE genes satisfied the condition to be found")}
   pval<-binom.test(sum(sel_num )-1,sum(com_num ),p=0.5,alternative='greater')
   return(list(com_num=com_num,sel=sel,sel_last=sel_last,step=step,sel_num
=sel_num ,consist_ratio=consist_ratio ,pval =pval))
   }
           }
```

```
## # delete those genes with different dysregulated direction between deg1 and deg2
and merge ###deg1 and deg2
union_deg<-function(deg1,deg2)
{
  com<-intersect(deg1[,1],deg2[,1])
  com1<-match(com,deg1[,1])
  common1<-deg1[com1,]
  com2<-match(com,deg2[,1])
  common2<-deg2[com2,]
  pair<-(common1[,3]==1&common2[,3]==0|common1[,3]==0&common2[,3]==1)
  com_part=common1[pair==FALSE ,]
  loc1<-deg1 [,1]%in%common1[,1]
  sel1_part<-deg1[loc1==FALSE,]
  loc2<-deg2[,1]%in%common2[,1]
  sel2_part<-deg2[loc2==FALSE,]
  union_deg<-rbind(sel1_part,sel2_part,com_part)
  return(union_deg)
  }


## An integrate program for the algorithm based on the reproducibility evaluation to
select ##reproducible DE genes
full_deg<-function(expR,expS,geneid,step1,top_num,pval,consist_th,method)
{ pair_deg<-NULL
  full<-NULL
  pair<-pair_select(expR,expS,geneid,top_num,pval,method)
  n=length(pair$select_pair)
  k<-0
  for (i in 1:(n-1))
    {  for(j in (i+1):n)
              {  k=k+1
                     deg12<-
deg_select(pair$select_deg[[i]],pair$select_deg[[j]],step1,consist_th)
                          deg21<-
deg_select(pair$select_deg[[j]],pair$select_deg[[i]],step1,consist_th)
                     pair_deg[[k]]=union_deg(deg12$sel_last,deg21$sel_last)
               if (n>=3) {
                          consist<-NULL
                             for  (s in 1:n){
                                     consist1<-NULL
                                          if (s==i| s==j) {next}
                                           else {
                                              consist1=inter_com_gene(pair_deg
[[k]],pair$select_deg[[s]])
```

```
consist<-rbind(consist,consist1$sel)
}
}
pair_deg [[k]]=consist
}
}
}
full <-pair_deg[[1]]
if (n>=3){
for (i in 2:k){
full <-union_deg(pair_deg[[i]],full)
} }
Rval<-expR[,pair$index[pair$select_pair,1]]
Sval<-expS[,pair$index[pair$select_pair,2]]
Rval_mean<-apply(Rval,1,mean)
Rval_mean=as.matrix(Rval_mean)
Sval_mean<-apply(Sval,1,mean)
Sval_mean=as.matrix(Sval_mean)
if ('PD'%in%method){
fullval<-Rval_mean-Sval_mean}
if ('PFC'%in%method){
fullval<-Rval_mean/Sval_mean}
fullval<-cbind(geneid,fullval)
com<-match(full[,1],fullval[,1])
full[,2]<-fullval[com,2]
return(list(full=full,select_pair=pair$select_pair,select_deg=pair$select_deg,all_pairs
=pair$all_pairs,index=pair$index))
}
```
